# Supplementary material for: Dynalign II: common secondary structure prediction for RNA homologs with domain insertions
Source: Nucleic Acids Res. 2014 Nov 21;42(22):13939–48. doi: 10.1093/nar/gku1172 (PMC4267632; doi:10.1093/nar/gku1172)
Supplement: SUPPLEMENTARY DATA [file supp_gku1172_nar-02021-z-2014-File012.zip › manual/javadoc/RNAstructure_java_utilities/package-frame.html]

RNAstructure\_java\_utilities


# RNAstructure\_java\_utilities

## Classes

- BorderBuilder
- BorderedButtonPanel
- FieldPanel
- FieldPanel.FilePanel
- FieldPanel.NumberPanel
- FileChooser
- FileField
- HTMLCheckBox
- ImageGrabber
- MacChecker
- NumberField
- NumberField.DoubleField
- NumberField.FloatField
- NumberField.IntegerField
- RadioButtonPanel
- RNAstructureMenu
- ScrollerPane
- SimpleDialogHandler
- ValueSelectionDialog

## Enums

- FileChooser.Types
